# "The Hospital" Medical Book Supplement, No. 1

**Published:** 1907-11-16

**Authors:** 


### "THE HOSPITAL" MEDICAL BOOK SUPPLEMENT.—No. I.

CONTENTS.

English Medical Literature - - - - - - - - - - - - 183
Surgical Literature in England - - - - - - - - - - - 184
Some Worthy Additions to the Practitioner's Library - - 186

### ENGLISH MEDICAL LITERATURE.

ENGLISH medicine has always been fortunate in possessing a few men of the highest mental qualities and of unblemished reputation. These men, educated in the earlier times at the courts of kings or in the houses of the great nobility, in later times at the universities, have been men of affairs as much as physicians, and their sound commonsense has helped them in the cure of disease more than the humoral theories to which they successively subscribed. English medical literature therefore is very varied in character. There is a mass of teaching, veiled for the most part in the decent obscurity of a learned language, which enshrines a mystic medicine based on astrology and distorted Galenic teaching, whose accessories, or rather actual working tools, were urinoscopy and horoscopes. There are books written in English, and afterwards translated into Latin to give them a wider circulation, and many of these books are immortal. Harvey's "Exercitatio," Sydenham's "Observationes Medicæ," and Sir Thomas Browne's "Religio Medici" are examples. Others, again, written in English, have remained to us as models of what medical treatises ought to be. The best of these *opera aurea* are Latham's "Lectures on Subjects connected with Clinical Medicine," Sir Thomas Watson's "Lectures on Physic," and Bright's "Reports of Medical Cases." There exists a fourth class, which is almost peculiar to English medicine—a class which contains some of the greatest literary triumphs of English physicians—books which are so entirely independent of medicine that it seems to be merely an accident that the author was a physician, and yet books which would probably never have been conceived but for the medical training of the author. Locke's "Essay on the Human Understanding"; Garth's poem, "The Dispensary," with all that it meant to Alexander Pope, and the poetry of Akenside and of Armstrong, will occur to everyone. Tobias Smollett and Sir A. C. Doyle are household words in this connection. Darwin's "Loves of the Plants" and Blackmore's works do not come into the same category; Arbuthnot's works have exercised a slighter influence on succeeding generations than the verdict of his contemporaries would have led us to expect.

Physicians for many years were the recognised teachers of the medical profession, and the lectureships to which they were appointed formed an important part of the income of many young men who afterwards became famous. Dr. Banester and Dr. Alexander Read were the best known of these teachers in London during the later part of Elizabeth's reign. Dr. Banester taught anatomy and surgery at the Barber Surgeons' Hall, and soon attained a great reputation. There exists a spirited picture, dated 1588, which shows him in the act of lecturing upon anatomy to an audience of Barber Surgeons. His lectures were afterwards collected and published in a volume which teaches us exactly the scope of contemporary knowledge. Dr. Alexander Read was the next great teacher of anatomy in London, and, like Banester, he lectured on surgery also. His lectures were published, and give us a very poor opinion of their matter, however able the exposition may have been. This monopoly of teaching by the younger physicians bore good fruit, for they acquired a familiarity with the details of dissection, and with the principles of physiology, which culminated after a few generations in the volumes of Harvey, Willis, Wren, Glisson, and Clopton Havers; whilst the work of Mayow was so far in advance of his time that it is only now beginning to receive adequate recognition.

Anatomical knowledge sometimes took curious byways, the best instance of which is Dr. John Smith's paraphrase upon the six verses of the twelfth chapter of Ecclesiastes, beginning "Remember now thy Creator in the days of thy youth." In a book of 266 pages, published in 1666, Dr. Smith labours to prove, and apparently satisfies himself, that King Solomon was an exact anatomist, and that these six verses contain an epitome of his knowledge. For Dr. Smith "the silver cord" is the spinal cord, and "the golden bowl" the pia mater; whilst he states roundly that this part of the allegory could never be understood before the time of Harvey, for "the pitcher" represents the veins. "The fountain" is the right ventricle of the heart, "the wheel" is the instrument of circulation, and "the cistern" is the left ventricle! The statements of this commentator are interesting, as they show the shifts to which previous exponents had been put when there was as yet no doctrine of the circulation. Thus Peter Lowe, who was a mere surgeon, had ventured to state in 1612: "The Pitcher broken at the fountaine: that is the great Vena Cava that may no more shoot blood from the liver, which is the spring that humecteth" (moisteneth) "the whole body in such sort that it serveth no more than a broken vessel. The wheel broken at the Cistern, that is the nerves and bladder doth grow so weak that they can no more retain water."

The work of the younger physicians in anatomy and physiology was unequal. They were still trammelled by authority, and their writings are a perfect labyrinth of theories, from which it is hard to disentangle facts. Sir Theodore de Mayerne is to some extent an exception. He published a series of cases which came under his notice between the years 1605 and 1640. The descriptions are excellent, the reflections minute and judicious, and the diagnosis and prognosis first rate. But to each case is appended a commentary in the shape of theories, and the vast farrago of medicines given or recommended leaves the reader in a state of mental confusion. Indeed, the general impression left after perusing his work is that a brilliant clinical physician and a man of great common-sense was lost owing to the theories of disease in which he had become involved. But for all this the cases form a most interesting series, and the illnesses of many persons who have since become historical detailed. The histories and methods of Sir Theodore (1573-1654) may well be compared with those of Sydenham, who lived a few years later (1624-1688). Mayerne gave abundance of drugs in every case; Sydenham would sometimes give no medicine at all, though usually he was not sparing: Mayerne was a courtier and man of the world, who often protected himself by routine and precedent; Sydenham's only rule seems to have been that "What is useful is good." It was said of him seriously by Blackmore, scoffingly by Gideon Harvey, that he made it his principle to go contrary to the practice of other physicians. Yet much may still be learnt from the writings both of Sir Theodore de Mayerne and of Thomas Sydenham by those who have the time and opportunity to read them. Medicine is more fortunate than surgery in that Dr. Freind has written a learned but very excellent "History of Physic," published in two volumes in the reign of George I. The history takes the form of a discourse written to Dr. Mead whilst the author lay, a political prisoner, in the Tower of London. Freind owed his release to Mead, who is said to have refused to prescribe for Sir Robert Walpole until the latter signed an order for his release. The History is well worthy of a more careful study than it has received latterly, for it reviews in a clear and easy manner the practice of physic from the time of Galen to the beginning of the sixteenth century.

The eighteenth century produced nothing very noteworthy in the way of medical literature in England, but the nineteenth century was prolific in good work. The new science of pathology first gave an impetus to the study of scientific medicine in the earlier years of the century, and Dr. Baillie came into notice first as an able teacher, then as a morbid anatomist, and lastly as a great physician. Morbid anatomy had hardly begun to exercise its full effect in converting medicine from an empirical into a scientific profession when Laennec, by his introduction of the stethoscope, gave a new generation of young physicians opportunities which they were not slow to turn to advantage, and enabled them to add greatly to medical literature in its best form. Medicine presented an attractive field, and for some years the best minds in the profession were attracted to physic rather than to surgery. The polish of the Harveian orations and the writings of Sir Henry Halford and of Sir Thomas Watson formed noble examples of English prose, which were long copied by those who aspired to maintain the prestige of English medical literature.

### SURGICAL LITERATURE IN ENGLAND.

The literature of English surgery may be said to have sprung all armed during the reign of Queen Elizabeth. Surgeons wrote but little before the Reformation, and, if we except the writings of John of Ardern in the fourteenth century, what they wrote was to very little purpose. Between 1560 and 1590 a number of books on surgery appeared for the first time written in English, books which were not only excellent in substance, but first-rate in style. The chief writers were Thomas Gale, William Clowes, and John Banester in London; John Halle at Maidstone; John Read at Gloucester; and Peter Lowe at Glasgow. All these writers knew something of each other, for all had served abroad in the military expeditions in France and Spain, and they were thus comrades in arms. They were united in a common desire to raise surgery from the degraded position which it then held, and from a trade to make it a profession. This desire led them to very fine flights of eloquence, and we owe to it many graphic details about the state of surgery during the Tudor period.

The dominant note which runs through the writings of these great surgeons is the wish to inculcate a very high standard of work and of morality in everyone who practised surgery, a standard which there is very little doubt they exemplified in their own lives. Gale expresses the sentiment in the following passage, which appears in an address to the younger surgeons:—"I pray you remember that ye be very studious in this art and diligent and neat in the practising thereof; and also to be modest, wise and of good manners and behaviour and that you lack none of those good properties that we have spoken of before, lest when you shall be called for in the time of necessity to serve princes and other noble persons, ye do not only dishonour yourselves and your country but this worthy art also. Remember, I pray you, what great charge is committed unto you in the time of wars. Ye have not only charge of men's limbs, but also of their lives, which if they should perish through your default, either in neglecting of anything that were necessary for their health which you ought to be furnished withal, either else through lack of knowledge which ye ought to have in your art—I say if these defaults be in you and the people perish in your hands you cannot excuse yourselves of your brother's death."

These Elizabethan sentiments were not peculiar to Gale and his companions, for they were re-echoed in the next generation by John Woodall, the principal surgeon to the newly-established East India Company, who wrote a book called "The Surgeon's

Mate,'' as a guide to young surgeons who were going to the East. '' The Surgeon's Mate '' is full of details of cases interspersed with much sound advice. It shows Woodall not only as a surgeon of experience and resource, but as a deeply religious man, thankful to God for the cures which his skill enabled him to make, and breathing the same spirit that enabled Ambroise Paré to write, '' I dressed him; God cured him.'' Here is such an extract: '' And as for my brethren of the younger sort, let me lovingly advise you neither for vain ostentation sake, nor popular applause, by rashness, to be guilty of the effusion of blood by unadvised amputation, though you may pretend you have Art for a sufficient warrant, or for a buckler; lest God touch your hearts for it in secret, who seeth not as men see, for the artificial shedding of blood hath no warrant nor encouragement written in God's book; wherefore in matters of weight be not too rash, but be advised by counsell.'' Woodall had travelled widely in Germany, Poland, and France. He had extensive experience in treating the plague, and he was attracted to London by the tremendous outbreak in 1603. It is no wonder, therefore, that his experience in plague, pestilence, and famine strengthened his reliance on Divine Providence, and made him write soberly and honestly, setting down what he knew in the plainest language for the benefit of others.

Woodall's book, published in 1639, marks the end of the first period of English surgical literature, a period characterised by detail and almost free from the modern spirit of inquiry or generalisation. The Elizabethan surgeons were contented to recount individual cases, to relate the effects of dressings discovered by themselves or handed down to them by their masters, and to advertise their practice in this, that, or the other branch of surgery, often with an unblushing effrontery which makes the reader smile to think how slight is the difference between writers of the sixteenth and of the twentieth centuries. The writings of these older surgeons record accurately and often graphically the cases they saw, but as yet they were not sufficiently educated to generalise upon their facts.

The second period of surgical literature is opened by Richard Wiseman, Serjeant-Surgeon to King Charles II., whose '' Chirurgical Treatises '' were published in 1686. Wiseman, like all the surgeons of his time, had seen much military service during the Civil War at home, and afterwards against the Dutch. He reflects faithfully a new epoch when the Royal Society had its beginning, when Sydenham was advancing physic, and the Oxford school was doing as much for anatomy. Wiseman was able to generalise; he narrates his cases with no less care than did the Elizabethan surgeons, but he compares one with another, and is able to deduce conclusions, which are sometimes wrong, but are often far-reaching and correct. By these means he brings surgery a step nearer to our present conception of a science to be studied, as well as an art to be practised. Like his predecessors, Wiseman is a man of sterling honesty, for he says: '' When the young chirurgeon shall find the cure easy in theory and appear so at first in the practice too, yet suddenly deceive him with a relapse, and not only once, but often delude his best endeavours; when the bystanders and persons concerned shall begin to accuse him of knavery in his proceedings and think him to pull back a cure, whilst he is rolling Sisyphus his stone, which will tumble down whether he will or not; he will then wish that all other practitioners had done what I have done in this treatise viz. recommend their observations both successful and unsuccessful, thereby increasing knowledge in our profession and leaving sea-marks for the discovery of such rocks as they themselves have split upon.''

Wiseman's method was followed and improved by many succeeding writers on surgery, until it reached its culminating point in the writings of Percivall Pott. Like Wiseman, Pott was of necessity a practical rather than a scientific surgeon, for pathology as yet had no existence. The descriptions of his cases are so clear, and the facts are so well stated, that it is generally possible to recognise their nature and to draw conclusions from them by the light of modern knowledge; whilst the cases narrated by many of his contemporaries and successors are incomprehensible from the manner in which theories are mingled with facts. Pott had the saving grace to know that surgery had not reached finality on the one hand, and that many of his predecessors were men of knowledge and honesty on the other. He says in his treatise on Fractures: '' I am very willing to allow that many parts of surgery are still capable of considerable improvement; and this part perhaps as much, if not more than any, it being one of those in which the observance of and rigid adherence to old prescribed rules have prevented the majority of practitioners from venturing to think for themselves and have induced them to go on in a beaten track from which they might not only safely but advantageously deviate.''

Pott wrote easily and well, but after his death there came a long period, fruitful indeed in good work but devoid of literature in the best sense of the word. The school founded by John Hunter too often followed their master in a slovenliness of expression which concealed a remarkable originality of thought, and compared most unfavourably with the older writers.

Sir Astley Cooper and Sir William Lawrence were brilliant exceptions. Both were men of high cultivation, able and willing to clothe their thoughts in clear and appropriate language. Sir James Paget followed these writers a few years later, and brought back the style of surgical writers to a very high level. For this style, as well as for their substance, his writings are worthy to be read and to be read again. So much is written at the present day that some authors are content not only to dictate their thoughts to an amanuensis but even to allow another hand to revise what has been sent to the press. It is impossible, under such conditions, to write anything that will be worthy of preservation on account of the manner in which it is written, and few surgical writers can hope to have their writings preserved for the matter they contain, because surgery is progressive and constantly changing. It appears, therefore, that the tendency of modern surgical literature is once more in the direction of bad style, which is rather the result of carelessness than of imperfect knowledge.

### SOME WORTHY ADDITIONS TO THE PRACTITIONER'S LIBRARY.

It would be manifestly impossible to pass in review all the various works in the different departments of medical science which have appeared, either as totally new works or as revised, corrected, and enlarged editions of older publications, during the past year. Nor do we believe that such a detailed enumeration of the additions to medical literature will have any real or lasting value to the practitioner. It is our intention to devote more attention to books which are worthy of inclusion in the practitioner's library, and with that limitation in view we have thought it advisable, at least in this our first book supplement, to confine ourselves strictly to works issued during the year by British publishing firms, and to pass by the many excellent volumes which have emanated from American and Continental houses. This limitation also necessitated a process of weeding out and selection from even the lists of British publishers. Highly specialised works, and, again, text-books obviously designed for the use of students preparing for examination, could not be included in our survey without extending the notice much beyond the limits of our space. What we have endeavoured to give, in the short *résumé* that follows of the year's output, is a brief notice of the main features in the way of novelties of interest to the general practitioner in the various publishers' catalogues and lists. During the past twelve months a large number of new books and new editions of old favourites have been issued; so many, indeed, that it would be impossible for us, in the comparatively short space available in our first book supplement, to give an adequate review of each volume. For a fuller and more comprehensive notice of the various medical and surgical works and treatises on special subjects here enumerated our readers are referred to back numbers of The Hospital or to future issues of The Hospital Medical Book Supplement.

### MEDICINE.

### Some New Books and New Editions.

The advent of Messrs. Hodder & Stoughton as medical publishers in association with Mr. Henry Frowde, of the well-known Oxford University Press, has been marked by the publication of several interesting and extremely excellent manuals. *The System of Medicine*, edited by Professor Osler and Dr. MacCrae, of which two volumes have seen the light, is a useful addition to the practitioner's library, and the Oxford Medical Manuals (among which we may specifically mention as worthy of the general practitioner's consideration, with a view to a permanency on the library shelves, Dr. Sutherland's *Treatment of Disease in Children*, Dr. Adamson's *Skin Affections in Childhood*, Dr. Poynton's *Heart Disease*, Dr. Gee's *Medical Lectures and Clinical Aphorisms* and *Auscultation and Percussion*, and Dr. Guthrie's *Functional Nervous Disorders in Children*) are specially written and eminently suitable for the practitioner, though they will doubtless be greatly appreciated by the senior student as well.

Messrs. J. and A. Churchill, 7 Great Marlborough Street, have issued a large number of new books and new editions during the year, and the volumes emanating from this well-known firm continue to show the high excellence of printing and general get-up which characterises all Messrs. Churchill's publications. In their list of new books appears the following: Dr. Hadley's *Nursing: General, Medical, and Surgical* (2nd edition), Lucas and Latham's *The Book of Prescriptions* (9th edition), Hale White's *Materia Medica* (10th edition), Starling's *Physiology* (8th edition), Hawk's *Practical Physiological Chemistry*, Dr. Wiley's *Foods and Adulteration*, *A Dictionary of Medical Terms—English, French, and German*, *A Dictionary of Medical Conversation, German-English*, Hewlett's *Pathology* (2nd edition), and Barlow's *Experimental Pathology* (2nd edition).

Messrs. Baillière, Tindall and Company's list is an equally long one, from which we select the following medical works: *A Dictionary of Medical Diagnosis* (McKisacks), Kraepelin's *Lectures on Clinical Psychiatry* (2nd edition), Gant's *Guide to the Examinations* (7th edition). Syme's *Bacteriology of Everyday Practice*, Somerville's *Practical Sanitary Science*, Sutherland's *Blood Stains*, and the very practical and excellent handbooks constituting their well-known "Student's Aid Series." Dr. Thorne's little work on *The Nauheim Treatment of Heart Disease* is an interesting exposition of the method of treatment which practitioners should read.

Messrs. Macmillan and Company's chief features have been the issue of the new volumes of Allbutt's *System of Medicine*, the second edition of Part II. of vol. ii. of which has recently been published, and the publication of the third edition of Dr. Hewitt's *Anæsthetics*. This firm has also published the second edition of Professor Allbutt's *System of Gynæcology*, and, as new books, Dr. Bruce's *Clinical Psychiatry* and Dr. Smith's *Differential Diagnosis*, both of which can be commended to the general practitioner.

Charles Griffin and Company, Exeter Street, Strand, have issued Dr. Samuel West's treatise on *Diseases of the Respiratory Organs*, in two volumes, a work which is of the greatest interest and will prove of the highest service to the general practitioner. In their list, also, are to be found the following new editions of well-known and popular text-books: Davies' *Handbook of Hygiene*, Reid's *Sanitary Engineering*, Blyth's *Foods*, Oppenheimer's *Toxines and Anti-Toxines* and *Ferments and their Actions*—two handbooks that should be on the library shelves of every practitioner who wishes to have at hand the best information on current theories of treatment. One of the most interesting volumes published by this firm is Dr. Robert Saundby's *Medical Ethics: a Guide to Professional Conduct*, the second edition of which has just appeared. It is a small and handy manual of medical etiquette—concise, clear, and practical, and it is one of the books with which the practitioner cannot afford to dispense. Another useful work is the second edition of Professor Pavlov's *The Work of the Digestive Glands*. The Pocket-book Series, of which new editions have appeared during the year (one of the latest being Dr. Brooks's *Tropical Hygiene*), are also issued by this firm, whose list in addition includes Dr. Dixon Mann's *Forensic Medicine and Toxicology*, the fourth edition of which is about to be issued; Von Jaksch and Garrod's *Clinical Diagnosis* (5th edition), one of the most valuable practical manuals; Dr. Bury's *Clinical Medicine* (2nd edition); Dr. Bevan Lewis's *Mental Diseases* (2nd edition); Sir T. McCall Anderson's *Diseases of the Skin* (2nd edition); Dr. Hunter's *Pernicious Anæmia*, and Dr. Brend's *Medical Jurisprudence and Toxicology*, a concise and readable exposition of the principles of medical jurisprudence.

Messrs. John Wright and Company, of Bristol, list several new works, among which we may specially mention *An Index of Treatment*, edited by Dr. Robert Hutchison and Mr. H. S. Collier. This is a manual which embodies, within a moderate compass and in a form extremely convenient for reference purposes the various methods of treatment for both medical and surgical disorders. Each article is a condensation admirably compiled, and the book should prove of real assistance to the practitioner who refers to it. Dr. Dampier Bennet's *Physical Methods in the Treatment of Heart Disease*, and the same author's *Re-education of Co-ordination by Movements* are also published by this firm.

To Mr. William Heinemann English practitioners owe one of the finest translations of German works published during the last few years. Of this the monumental *Metabolism and Practical Medicine* of Professor Carl van Noorden two volumes were published early in the year, the final and third volume being issued recently. The translation is the work of several collaborators under the able editorship of Dr. Walker Hall, and the work is one which at the time of issue we cordially welcomed. Mr. Heinemann also published Professor Chittenden's Lectures on Dietetics (*The Nutrition of Man*), and announces a translation of Professor Elie Metchnikoff's book, *The Prolongation of Life*, by Dr. P. Chalmers Mitchell. Messrs. William Green and Sons, of Edinburgh, published Dr. W. E. Fothergill's *Manual of Midwifery*, a useful handbook for the student and practitioner.

Among other medical works which have appeared during the year and which merit the appreciative consideration of the general practitioner may be mentioned the following: Muir and Ritchie's *Bacteriology* (Young J. Pentland, Edinburgh), the fourth edition of which has just been issued maintaining the high standard of excellence which was shown by the first and later editions. Dr. Luff's *Gout: Its Pathology and Treatment* (Cassell and Company, London), the revised and enlarged third edition of which has just appeared; Martindale's *Extra Pharmacopœia*, the new twelfth edition of which has been published by Mr. H. K. Lewis, of 136 Gower Street, W.C.; Savage and Goodall's *Insanity*, the revised fourth edition of which has lately been issued by Cassell and Company; Manson's *Tropical Diseases*, of which a new edition is announced by the same firm; Messrs. Cassell and Company's new series of text books on "Modern Methods of Treatment," of which the following have already appeared; Dr. Bosanquet's *Serums, Vaccines, and Toxines*; Dr. Burton Fanning's *Open-air Treatment of Pulmonary Tuberculosis*, and Dr. Batty Shaw's *Organo-therapy*—all books which should have a place in the up-to-date library.

Mr. Henry Kimpton published lately *The Practice of Obstetrics*, edited by Dr. Reuben Petersen, a bulky volume of 1,087 pages, containing much useful and valuable information—a book which is likely to be of service to the general practitioner. A companion work by Dr. Bovee, entitled *A Practice of Gynæcology*, was issued earlier in the year, and another volume which the same firm published was Dr. Koplik's *The Diseases of Children*, a very full and exhaustive review of pediatrics.

Mr. Sidney Appleton issued Dr. Torcheimer's large manual on *The Prophylaxis and Treatment of Internal Diseases*, a work which was favourably received by the profession, and which will repay perusal by the general practitioner, although its teaching is in some points not quite in a line with that of other text-books. The sixth edition of Professor Osler's well-known text-book of medicine was also issued by this firm. Rebman, Limited, were responsible for Dr. Jackson's *Tropical Medicine*, a useful handbook; Dr. Braddon's *Cause and Prevention of Beri-Beri*, another valuable addition to the library of the student in tropical diseases; and Dr. Greene's *Medical Diagnosis*. The fourth edition of the deservedly popular *Dictionary of Treatment*, by Sir W. Whitla, was published by Mr. Henry Renshaw, 356 Strand. The new edition is one of the fullest and best manuals of treatment on the market, and the practitioner in doubt as to which book on the subject to buy would do well to consider the merits of Whitla very carefully before he makes up his mind. An interesting, suggestive, and instructive little book, the joint work of Mr. Barton and Mr. Gresswell, was published early in the year by Everett and Company, 42 Essex Street, under the title of *Elements of the Practice of Comparative Medicine*. Mr. H. J. Glaisher reissued Dr. George Herschell's well-known works on *Indigestion and Constipation*, and also published a second edition of the same author's little treatise on *The Diagnosis of Gastric Carcinoma*, a clearly-written summary of the essentials of the disease, which will be of use to the practitioner. The fifth edition of that excellent condensation of *The Diseases of Children*, by Dr. Ashby and Mr. Wright, a book which no general practitioner ought to omit from his library catalogue—was issued by Longmans, Green and Company, the same firm also republishing, for the fifth time, Dr. Coats' *Manual of Pathology*, thoroughly revised and brought up to date by Dr. Sutherland. Dr. Roberts' *Theory and Practice of Medicine* has run into a tenth edition, which is issued by Mr. H. K. Lewis, who also publishes a fourth edition of Dr. Buxton's *Anæsthetics*. Both books are so well known that it would be superfluous to enumerate their good points.

Bale, Sons, and Danielsson have just issued the second edition of Dr. Daniel's *Laboratory Studies in Tropical Medicine*, a handy, practical manual for the student in tropical diseases. The book is well illustrated, and is fully up to date.

Messrs. G. P. Putnam's Sons have recently issued the second edition of Dr. Herter's *Diagnosis of Organic Nervous Disease*. This is a comparatively short manual, but it is likely to prove of great service to the practitioner, as it is one of the clearest and most succinct of text-books on the diagnosis of nervous diseases we possess.

Mention must be made of the new *British Pharmaceutical Index*, published by the Pharmaceutical Society at 72 Great Russell Street, London, W.C. This is indeed what it claims to be on the title page, "an imperial dispensatory for the use of medical practitioners and pharmacists," and it is a work that should have its place on the library shelves within handy reach, for its usefulness as a reference manual is unquestionable.

Messrs. Archibald Constable & Co., Ltd., republished Professor Starling's *Recent Advances in the Physiology of Digestion*, a small work which, although of special value to the student preparing for higher examinations, will be of use to the practitioner as well. This firm also issued Messrs. Battle and Corner's *Surgery of the Appendix Vermiformis*, a valuable addition to the literature of appendicitis; Morat's *Physiology of the Nervous System*, translated by Dr. Syers; Messrs. Dudgeon and Sargent's *The Bacteriology of Peritonitis*, a work from which the practising surgeon will derive much useful information.

### SURGERY AND SPECIAL SUBJECTS.

Among the new works published in this country on surgery there are few of outstanding merit. There have been several very excellent new editions of old favourites which cannot be passed by without a word or two of appreciative comment, but of the new works themselves it cannot be said that they have fully justified their appearance. There are so many good editions of standard surgical text-books that a new manual of general surgery suffers inevitably from comparison and contrast with old and established friends whose claims have become to be tacitly admitted by most students and practitioners.

Starting with the new books, the latest in the field is *A Manual of Surgery*, by Francis T. Stewart, M.D., Professor of Surgery at the Philadelphia Clinic, a large crown octavo book of 778 pages, published by Rebman, Limited, 129 Shaftesbury Avenue, London. Dr. Stewart's reputation as a bold and fearless surgeon will win for him a large number of readers, and though we do not believe that his text-book will supersede any of our standard manuals of surgery, we are convinced that most general practitioners will find it helpful and serviceable as an adjunct to their Holmes, Walsham and Spencer, or Rose and Carless. The surgical volumes of the Oxford Medical Manuals (Henry Frowde, Oxford University Press, and Hodder and Stoughton, Warwick Square, E.C.) are short monographs on special departments and are not intended to replace the usual text-books. All of them are excellent in style and printing—with the exception of a few typographical errors, which are easily rectified in subsequent editions—but they vary in value and usefulness. The general practitioner will find Mr. Eldred Corner's *Diseases of the Male Generative Organs* and *Operations in General Practice*, Mr. Tod's *Diseases of the Ear*, Mr. Wagget's *Diseases of the Nose and Throat*, Mr. Wallace's *Enlargement of the Prostate*, and Mr. Parson's *Pathology of the Eye* fully worthy of a place on the library shelf. Mr. Lockwood's excellent surgical lectures, the third edition of which has recently been issued in the same series, is too well known and too widely appreciated to need more than passing mention. Mr. Parson's *Diseases of the Eye* (Churchill), and Mr. Cheatle's *Points in the Surgery of the Temporal Bone* (Churchill) may also be mentioned. Messrs. J. and A. Churchill have also during the year issued new editions of recognised standard text-books. First of these is the fourth edition of the well-known treatise on anatomy, edited by Mr. Henry Morris. *Morris* is so well known that it would be a work of supererogation to dilate on the excellence of this revised and enlarged edition, which in its present form is undoubtedly one of the finest text-books on the subject in the English language. Mr. Jacobson's *The Operations of Surgery* is almost as well known, and the fifth edition of this popular work, in which the author has collaborated with a new writer on surgical methods—Mr. R. P. Rowlands—has just been issued. It is much more bulky than its predecessors and contains, especially in the second volume, much that could usefully have been condensed with benefit to the reader, but on the whole it well maintains the excellent quality of superiority which characterised former editions. A third favourite, re-issued by this firm is the fifth edition of Mr. Bowlby's little work on *Surgical Pathology*. The same firm also issued new editions of Mr. Harrison Cripp's well-known manuals *Cancer of the Rectum* and *Diseases of the Rectum*, and announces a new edition of Mr. Hurry Fenwick's *Cardinal Symptoms of Urinary Disease*, and a new work by the same author—*X-Ray Diagnosis of Urinary Stone*.

Messrs. Baillière, Tindall and Cox have published several interesting works during the year. Among these may be mentioned Moorhead's *Surface Anatomy*, Wheeler's *Student's Handbook of Operative Surgery*, Walsh's *Röntgen Rays*, and the little works on surgical subjects included in their condensed "Student's Aid Series." Dr. Giles' *Gynæcological Diagnosis* is a book which we can confidently introduce to the practitioner's notice. It is clearly written, admirably lucid, and eminently suitable for the working practitioner as opposed to the student reading merely for examinations. Mr. Bruce Bennie's work on *Hip Disease*, Mr. Edwards' on *Carcinoma of the Rectum*, Mr. Wallis's larger work on *The Surgery of the Rectum*, Mr. Lake's *Handbook of Diseases of the Ear*, and Mr. Bidwell's *Handbook of Intestinal Surgery* are all "practitioners' books," one or all—preferably all—of which should figure on the library shelves. The same firm also issued the two volumes of Buchanan's *Manual of Practical Anatomy*, which is one of the best dissecting-room manuals the student can buy, while to the general practitioner its usefulness is far above that of ordinary works on practical anatomy (which are usually only designed for actual workers on specific parts). W. B. Saunders and Company have issued Heisler's *Embryology* (3rd edition) and the second edition of Eisendrath's *Clinical Anatomy*. From the well known Edinburgh publishing house of Messrs. Young J. Pentland emanated the second edition of Miles and Thomson's *Manual of Surgery*, in two volumes, an extremely readable, handy, compact, and up-to-date treatise of surgery for students' use. From the same publishing house came a very readable treatise on *Intussusceptions*, written by Mr. Charles Clubbe and giving the writer's experiences in 144 cases—a valuable addition to the list of works on this important subject. Cassell and Company issued new editions of Mr. Bland Sutton's well-known work on *Tumours, Innocent and Malignant*, Mr. Pearce Gould's *Surgical Diagnosis*, Dr. Herman's *Diseases of Women*, and Sir Frederic Treves' *Surgical Applied Anatomy*, and announce a new edition, soon to be published, of the last author's popular *Manual of Operative Surgery*. Mr. H. K. Lewis published a third edition of Mr. Binnie's *Manual of Operative Surgery*. Brown and Sons, Limited, 5 Farringdon Avenue, E.C., issued a small volume on *Modern Methods for Securing Surgical Asepsis*, from the pen of Mr. Edward Harrison—a readable little treatise on the essentials of asepsis. Messrs. Macmillan announced Mr. Ballance's volume on *Some Points on the Surgery of the Brain and its Membranes*.

Mr. H. K. Lewis published the ninth edition of Sir Henry R. Swanzy's *Handbook of Diseases of the Eye and their Treatment*, a work that is in every way worthy of the popularity it has won, and the appreciation which it has received since the first edition was issued.

Messrs. John Wright and Company, Bristol, published a translation, by Mr. Montsarrat, of Professor D. Schlesinger's *Indications for and against Operation in Diseases of the Internal Organs*, a small book that will prove of great use to the busy general practitioner. The same firm also issued Dr. Wilson's *Ingleby Lectures on Pelvic Inflammation in the Female*. Messrs. Longmans, Green and Company, 39 Paternoster Row, published the sixteenth edition of *Gray's Anatomy*, thoroughly revised and greatly enlarged—a volume which is quite capable of holding its own against the many excellent manuals of anatomy designed for the use of practitioners as well as students. John Bale, Sons, and Danielsson published Mr. Reginald Harrison's new volume, *The Urethrotomies*, a collection of clinical lectures, written in the author's clear and lucid style, on subjects on which Mr. Harrison is an acknowledged authority. The same firm also issued the second edition of Mr. Garry Simpson's *Adenoid Growths of the Naso-Pharynx*, a small manual giving a synopsis of signs, symptoms, and treatment, which should be of great value to the practitioner who has not had much experience in "Throats." Two volumes on *Bodily Deformities*, a series of lectures delivered at the City Orthopædic Hospital, by the late Mr. E. Chance, appear on Messrs. Smith, Elder and Company's list. The first volume, edited by Mr. John Poland, is an extremely well written and thoroughly useful work, from which the practitioner may glean much helpful advice and instructive information. The same firm issued a revised sixth edition of Mr. Arnot Lawson's *Diseases and Injuries of the Eye*, one of the most reliable text-books of ophthalmology. Mr. Leedham Greene's monograph on *The Treatment of Gonorrhœa in the Male* was published by Messrs. Baillière Tindall and Cox, in the shape of a small but well-written and exceedingly concise manual, and Mr. Sidney Stephenson's Middlemore Prize Essay on Ophthalmia neonatorum was published by Messrs. Pulman and Sons. Messrs. Kegan Paul, Trench and Trübner published Messrs. Grimsdale and Brewerton's *Text-book of Ophthalmic Operations*, a work designed more especially for the general practitioner and the senior student. From Messrs. Saunders and Co. emanated the fifth edition of Da Costa's *Surgery*, and Dr. Webster's *Diseases of Women*.

